# Nitrate of Silver as a Therapeutic Agent

**Published:** 1894-08

**Authors:** 


ARTICLE II.

### NITRATE OF SILVER AS A THERAPEUTIC AGENT.

The following is the manner in which Dr. E. A. Stebbins, of Shelbourne Falls, Mass., recommends the application of argenti nitras for the prevention of recurrence of decay, as well as on account of a few cases and the results obtained:

### MANNER OF APPLICATION.

Make, of hard wood, fine, slender points that will enter very small cavities.

Put these points into handles on different angles suitable to reach all portions of the teeth (two points, one on an acute and one on an obtuse angle, will be sufficient).

Pulverize the crystals (owing to impurities in the common lunar caustic sticks, it is much preferable to use the crystals).

The salts are dissolved in an equal amount of water; therefore there should be but little moisture in the cavity, or on the surface to be treated.

Moisten the wood point a very little, so the powder will stick to it, and then take upon it an amount about the head of a common pin, or more, according to the size of the cavity or surface, and apply to every part of the diseased portion. Apply enough salts and moisture to be sure the whole surface is touched. The salts will take effect in a minute or so.

Waste amalgam scraps rubbed over the treated surface or cavity will take up the liberated nitric acid and turn the decay dark instantly. (Since writing this paper I have used silver filings.)

I have not sufficient data to determine whether the application of the amalgam is beneficial or not, but theoretically I think it is.

Silver instead of wood points may be used.

Of course the mouth of the patient should be protected during the operation.

Any slight touch to the tongue or other parts of the mouth will do no harm. I never heard a complaint of bad after results. Some dislike the taste.

Use colored napkins, so the stains will not show.

Do not allow the patient to wipe the mouth immediately with a handkerchief for fear of getting it stained.

After the salts have taken effect; and you are through with the treatment, at once inject a copious amount of water to carry away the surplus; also allow the patient to rinse the mouth well.

The *manner* of protecting the patient's mouth from being touched with the salts can be determined readily by each operator. Caution and experience will enable anyone to protect the patient's mouth and his own fingers.

SOME POINTS.

Nitrate of silver forms with albumen and fibrin definite compounds, that are insoluble, except with a few substances.

It is superficial in its action.

With the phosphates it forms nitrate of lime and phosphate of silver.

With the carbonates it forms carbonate of silver and nitrate of lime.

Superficial decay in labial and buccal surfaces show most favorable results.

Small cavities are more favorable than large ones.

If decay has reached the pulp, it is not safe to apply the silver.

In my experiments thus far I have not removed any decay before applying the salts.

Where the gum has receded, and the exposed cementum is sensitive, the effect is very beneficial. In such cases it seems to stimulate the gum to more healthy action.

The liberated nitric acid should be removed.

When asked by patients what the treatment does, I often tell them it kills, embalms, and buries the microbes right in the place where it finds them.

The following patients will now be presented:

Case 1.—Girl. The little pits in buccal surfaces of second lower temporary molars were treated in March, 1886. The apex cavities in upper temporary molars treated in September, 1890. None of these cavities seem to have decayed since treatment.

Case 2.—Boy. The buccal surfaces of temporary molars were treated March, 1886, and have not decayed since. The apices in lower temporary molars were treated in June, 1887, and, having begun to decay again, were retreated in November, 1889, since which time they seem to have kept quite well.

Case 3.—Girl. This patient had some cavities treated in 1888 and 1889, which you will observe are in a good state of preservation; while surfaces that appeared sound when the others were treated have large cavities now.

Case 4.—Girl. This patient had several cavities treated in 1888. A year after nearly all traces of the silver had disappeared, and decay was active. They were then retreated, and also some new cavities. Two years later all traces of the treatment had disappeared. Please observe that this girl is very nervous and of slight figure,—just the type of patients that often return to us for filling and refilling.

Case 5.—Man. This patient had six small cavities treated in apex surfaces of lower incisors in January, 1886, and have not been touched since. Please observe that the characteristic results of the treatment are perfect, and the decay has been entirely arrested.

Case 6.—Young lady. In 1888, when fourteen years old, she had nineteen cavities treated in the upper teeth,—most of them very large,—some adjoining fillings. Her health and nervous conditions were such that she could not have fillings put in, and must have relief in some way or lose her teeth. Present condition: First molars decayed all away. Three cavities that were treated have since been filled. The remaining cavities have the characteristics of the treatment, and seem not to be decaying.—*Ohia Journal of Dental Science.*

